# An Oxalato-Bridged Cu(II)-Based 1D Polymer Chain: Synthesis, Structure, and Adsorption of Organic Dyes

**DOI:** 10.3390/polym16121742

**Published:** 2024-06-19

**Authors:** Fouzia Munawar, Muhammad Khalid, Muhammad Imran, Muhammad Naveed Qasim, Shazia Waseem, Murad A. AlDamen, Muhammad Ashfaq, Muhammad Imran, Muhammad Nadeem Akhtar

**Affiliations:** 1 Department of Chemistry, Khwaja Fareed University of Engineering & Information Technology, Rahim Yar Khan 64200, Pakistan; fouziamunawar5@gmail.com; 2 Division of Inorganic Chemistry, Institute of Chemistry, The Islamia University of Bahawalpur, Bahawalpur 63100, Pakistan; muhammad.imran@iub.edu.pk (M.I.); n.qasim5122@gmail.com (M.N.Q.); w.shazia@yahoo.com (S.W.); 3 Department of Chemistry, School of Science, The University of Jordan, Amman 11942, Jordan; maldamen@ju.edu.jo; 4 Department of Physics, University of Sargodha, Sargodha 40100, Pakistan; ashfaq.muhammad@uos.edu.pk; 5 Research Center for Advanced Materials Science (RCAMS), King Khalid University, P.O. Box 9004, Abha 61413, Saudi Arabia; imranchemist@gmail.com; 6 Chemistry Department, Faculty of Science, King Khalid University, P.O. Box 9004, Abha 61413, Saudi Arabia

**Keywords:** Cu(II) coordination polymer, mixed ligands, crystal structure, dye sorption

## Abstract

In the current research, we prepared a polymeric framework, {[Cu(C_2_O_4_)(C_10_H_8_N_2_)]·H_2_O·0.67(CH_3_OH)]}_n_ (**1**) (where C_2_O_4_ = oxalic acid; C_10_H_8_N_2_ = 2,2-bipyridine), and explored this compound for adsorption of methylene blue (MB) and methyl orange (MO). The crystal structure of the compound consists of a Cu(ox)(bpy) unit connected via oxalate to form a 1D polymeric chain. This polymeric chain has adsorption capacities of 194.0 and 167.3 mg/g for MB and MO, respectively. The removal rate is estimated to be 77.6% and 66.9% for MB and MO, respectively. The plausible mechanisms for adsorption are electrostatic, π-π interaction, and OH-π interaction for dye stickiness. The adsorbent surface exhibits a negative charge that produces the electrostatic interaction, resulting in excellent adsorption efficiency at pH 7 and 8. The pseudo-first-order kinetic model is selected for the adsorption of MB and MO on the adsorbent. The reported compound has remarkable efficiency for sorption of organic dyes and can be useful in wastewater treatment.

## 1. Introduction

Rapid industrialization, population development, and the use of huge amounts of various dyes are the major contributors to rising water and environmental pollution [1,2]. Organic dyes are widely explored in various industries like plastic, rubber, paint, cosmetics, paper, and leather, and most of these industries discharge their effluents directly into the water resources, which causes harmful pollution. These types of dyes are hard to eliminate from an aqueous system due to their xenobiotic properties and aromatic structures [3,4]. Obtaining a model system to understand the type of organic dye decomposition is crucial, and various approaches such as adsorption, photo-catalytic degradation, membrane separation, chemical oxidation, coagulation, biodegradation, etc. have been explored [5,6]. Out of these various approaches, adsorption is low-cost, highly efficient, rapid, cheap, and has good recyclability [7,8,9,10]. In the literature, different traditional adsorbents like clay, activated carbon zeolites, resin, and porous materials have been widely used to adsorb these dyes [11,12,13] but mostly these are time-consuming and have weak adsorption capacities [14]. Hence, it is necessary to develop effective and economical adsorbents.

Nowadays, researchers are attracted to coordination chemistry-based discrete complexes due to various types of possible interactions [15], and coordination polymers and metal organic frameworks that have tunable porosity, ultrahigh surface area, ionic framework, availability of active sites, and stability provide a supervisory idea for the removal of dyes [16,17,18]. Further, according to the features and size of the adsorbate, coordination compounds have proper adsorption sites embedded onto ligands, and, additionally, various pore sizes can be indented and altered by changing the connected organic linker, therefore showing tremendous promise in applications for adsorption [19,20]. In the preparation of coordination polymers with diversity in functions, the choice of ligands with N- and O-donor sites plays a remarkable role [21,22,23]. In addition, various types of common intermolecular interactions, like π-π stacking and hydrogen bonding, also have significant effects on dye adsorption processes [24,25]. Moreover, time, pH control, counter-anions, and temperature also have influences on the adsorption of dyes [26].

Among various organic dyes, MB and MO are azo dyes, which are thought to be highly toxic and mutagenic to living things [27,28]. Coordination compounds have the capability to adsorb anionic dye (methyl orange) because of the effect of charge-selectivity [29]. Correspondingly, researchers used various coordination compounds for the adsorption of MB and MO dyes, respectively, efficiently [30]. MB also has potential applications as dying materials for cotton, wood, and silk [31], and MO is used in paper, food, textiles, printing, pharmaceuticals, and analytical laboratories [32]. Both of these dyes have adverse effects on health even in low concentrations and cause various adverse effects such as cyanosis, jaundice, quadriplegia shock, cyanosis, vomiting, quadriplegia, heart rate, jaundice, and necrosis in humans’ bodies [33]. Therefore, it is necessary to prepare suitable materials for the elimination of MB and MO from wastewater.

**Figure 1:**
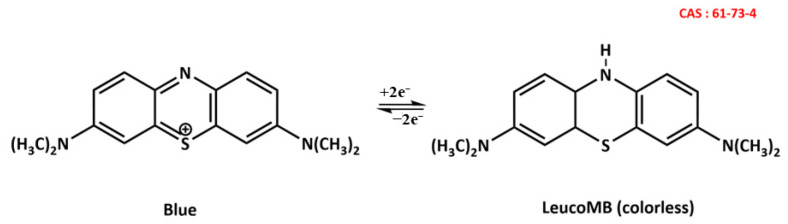
Structural representation of MB dye.

**Figure 2:**
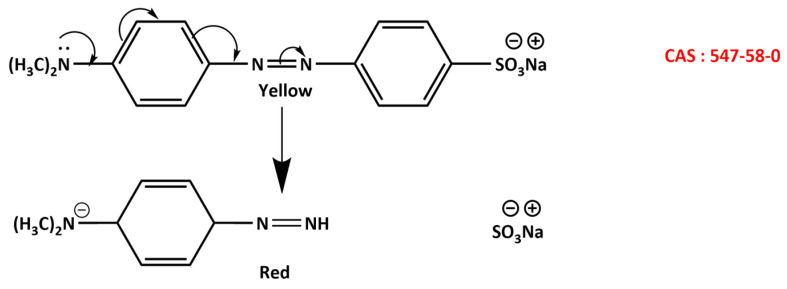
Structural representation of MO dye.

In light of the above considerations, our aim is to prepare effective, environmentally friendly, stable, and cheap materials for the removal of these dyes. Recently, we explored the Cu(II) complex with photocatalytic degradation of MB [34] and the Zn(II)-based coordination polymer for removal of MB [35]. In the present study, we report Cu-containing 1D polymer chains derived from oxalic acid and bipyridine and characterized through FTIR, elemental analysis, and the single X-ray diffraction method. Further, the thermal study and its applications to adsorb toxic organic dyes have also been investigated. The structural representations of MB and MO dyes are represented in Figure 1 and Figure 2.

## 2. Materials and Methods

Copper acetate monodydrate (CAS: 6046-93-1, 99.99%), oxalic acid (CAS: 144-62-7, 99.999%), 2,2′-bipyridine (CAS: 366-18-7, 99%), sodium azide (CAS: 26628-22-8, 99.5%), ethanol (CAS: 164-17-5, 99.45%), methylene blue (CAS: 61-73-4), and methylene orange (CAS: 547-58-0) were purchased from Sigma-Aldrich (Steinheim, Germany). Elemental % composition was measured through the Dumas method on ELEMENTARY UNICUBE^®^ IZI equipment (C, H, O, N, S). FTIR spectra were recorded on a Bruker Tensor 27 FTIR Spectrometer (Bruker, Billerica, MA, USA) by using KBr pellets. Thermal gravimetric analysis was carried out in a temperature range of 25–1000 °C, increasing by 20 °C min^−1^ on a Perkin Elmer Pyris Diamond instrument (PerkinElmer, Hopkinton, MA, USA).

### 2.1. Crystallographic Measurements

The single crystal data were collected on an Apex IV (Bruker) diffractometer equipped with a Mo Kα X-ray (λ = 0.71073 Å). The data reductions were performed using Bruker APEX4 software, and the absorption correction was conducted with the multi-scan SADABS method [36]. The refinement of the structure was executed by Olex2 [37] and the implemented ShelXT [38]. CCDC No = 2340883 (**1**) contains the supplementary crystallographic data, which can be obtained free of charge from the Cambridge Crystallographic Data Center via www.ccdc.cam.ac.uk/data_request/cif (accessed on 17 March 2024).

The crystallographic data and bond angles/bond lengths for **1** are presented in Tables S1 and S2 (Supplementary Materials), respectively.

### 2.2. Thermal Analysis

Thermal stability analysis of **1** was performed, and the TGA curve is depicted in Figure S1 (Supplementary Materials). It was observed that compound loss weight in multi-steps, such as the first curve, shows evaporation of volatile substances. There is a decrease in mass of 8.9% at 62.4 °C, which indicates the removal of methanol molecules. The next curve is obtained at 130 °C with a loss of 13% mass of the compound that indicates the removal of non-coordinated water molecules (with the accompanying endothermic peak on the DTA curve at 140 °C). Further, the mass decreases at 300 °C (334 °C at DTA) with a mass loss of 41.2%, which is related to the decomposition of 4,4, bipyridine. At 480 °C, there is a further reduction in mass, which shows the removal of oxalate, and finally, at 895 °C, only 16.56% of the compound is left, which is a residue of CuO. The thermal behavior of **1** is comparable with the published Cu oxalate containing coordination compounds [39,40].

### 2.3. Adsorption Batch Experiments

To measure the adsorption and removal efficiency of the Cu polymeric chain for the removal of MB and MO, an adsorptive study was performed.

First, an adsorption capacity of **1** was analyzed by changing the pH. The change in pH was adjusted with 0.1 M NaOH and HCl. Further, 10 mg of adsorbent (**1**) were dissolved in 250 mL of a solution of 0.01 mg/mL MB and MO, and pH was adjusted with 0.1 M NaOH and HCl. Then, 5 mL of solution was taken after each 10 min, and the MB and MO concentrations in the filtrate were calculated on a UV-vis spectrophotometer at 664 nm (max) and 464 nm, respectively. Adsorption equilibrium was formed after 60 min. The experiment was performed at pH 2, 3, 4, 5, 6, 7, 8, 9, and 10, respectively. The maximum removal efficiency shown by the compound was at pH = 8. This is because MB, being a cationic dye, shows adsorption in the low basic region. This is the optimum pH for further experimental parameters. The concentration of MB and MO is changed from 0.01 to 0.03 mg/mL, while the volume of the water (solution) taken is 250 mL with a one-hour contact time. The effect on concentration was estimated by continuous stirring, and the maximum adsorption was shown by **1** when the dye concentration was 0.01 mg/mL. Later, the investigation of the adsorption behavior of the compound was carried out by changing the temperature from 298 K to 318 K. The optimum temperature was 298 K due to the larger capacity and efficiency of the exothermic reaction. The maximum adsorption capacity and removal efficiency are shown by **1** for MB and MO when the dye concentration was 10 ppm, pH = 8, temperature = 298 K, and adsorbent dose = 10 mg, which is used as an optimum condition for further experimentation. Using the Beer–Lambert law, a (extinction coefficient) value was determined from the UV-vis findings of standard solutions [41]. The adsorption and removal abilities of **1** for MB were calculated by Equations (1) and (2), respectively [42].

(1) $$q=\frac{(C_{o}-C_{e})V}{m}$$

(2) $$R=\frac{\left(C_{o}-C_{e}\right)}{C_{e}}\times 100$$

where q = adsorption strength (mg/g), C_o_ = initial concentration (MB) (mg/L), C_e_ = equilibrium concentration of MB (mg/L), V = volume of solution (L), m = adsorbent amount (g), and R = removal efficiency or percentage efficiency.

Adsorption kinetics were investigated by introducing 20 mg of adsorbent (**1**) to a 500 mL flask with 250 mL of MB and MO (10 ppm) solution (pH 8). Magnetic stirring was used to agitate the flask for time intervals up to 60 min. After a specific interval of time, for the calculation of the change in concentration at wavelengths 664 nm and 464 nm, 5 mL of sample was collected, which was further utilized for the determination of removal efficiency and adsorption capacity using Equations (1) and (2). Various kinetics models, pseudo-first-order kinetics (Equation (3)), pseudo-second-order kinetics (Equation (4)), [43,44], and a liquid film diffusion model (Equation (5) [45]), were used for the experimental data.

(3) $$K_{1}=\frac{\ln q_{e}-\ln(q_{e}-q_{t})}{t}$$

(4) $$K_{2}=\frac{q_{t}}{t}(\frac{1}{q_{e}^{2}}+\frac{t}{q_{e}})$$

(5) $$k_{f}=\frac{A-\ln\left(1-\frac{t}{q_{e}}\right)}{t}$$

where pseudo-first-order kinetics rate constant is described by K_1_, pseudo-second-order kinetics rate constant symbolized by K_2_, while q_e_ = adsorption at equilibrium, q_t_ = adsorption at time t (mg/g).

To research pseudo-first-order, pseudo-second-order, and liquid film diffusion kinetic models, graphs are plotted between ln(q_e_ − q_t_) vs. time (t), t/q_t_ vs. time (t), and ln(1 − q_e_/q_t_) accordingly. Meanwhile, the models’ other parameters are computed by slope and intercept values. The values of R^2^ were computed by Origin 8.0 Software’s linear fitting of plots, as was mentioned in the previous paragraph. The adsorption capacity of **1** is 194.039 and 167.28 mg/g, and the removal ability was evaluated at 77.6% and 66.9% for MB and MO, respectively.

The adsorption isotherm was investigated by doing a concentration-shift experiment with 20 mg **1** (at 10, 20, and 30 ppm). The contact time for each solution was 1 h. Each flask (10, 20, and 30 ppm) was emptied and analyzed for concentration. Equation (1) was used to calculate adsorption capacity from the known values of the beginning and final concentrations. Adsorption models such as Langmuir (Equation (6)) and Freundlich (Equation (7)) were used to analyze the experimental data.

(6) $$\frac{C_{e}}{q_{e}}=\frac{C_{e}}{q_{max}}+\frac{1}{q_{max}K_{L}}$$

(7) $$\ln q_{e}=\frac{1}{n}\ln C_{e}+\ln K_{F}$$

where C_e_ = concentration at equilibrium (mg L^−1^), q_e_ = adsorption at equilibrium (mg g^−1^), K_L_ = adsorption constant for Langmuir (L mg^−1^), q_max_ = maximum adsorption capacity (mg g^−1^) and K_F_ n = Freundlich adsorption constant.

For studying the Langmuir and the Freundlich graphs are plotted between C_e_/q_e_ vs. C_e_ and ln(q_e_) vs. ln(C_e_), respectively, while their other parameters were estimated from intercept and slope values. The R^2^ values were found by linearly fitting their plots. 

### 2.4. Synthesis of {[Cu(C_2_O_4_)(C_10_H_8_N_2_)]·H_2_O·0.67(CH_3_OH)]}_n_ (***1***)

A mixture of Cu(II) acetate monodydrate (0.50 mmol, 0.10 g), oxalic acid (1.0 mmol, 0.10 g), 2,2′-bipyridine (1.0 mmol, 0.15 g), and sodium azide (1.0 mmol, 0.06 g) in EtOH/H_2_O (1:1 ratio) was sealed in a Teflon-lined autoclave and kept for reaction at 150 °C for 48 h. Blue block-shaped crystals were collected. Yield: 46% (based on Cu). Elemental Analysis (%) for C_13.34_H_13.33_CuN_2_O_5.67_: Calculated: C = 45.02, H = 3.77, and N = 7.87. Found: C = 44.95, H = 3.71, N = 7.69 FT-IR (cm^−1^): 3764 (w), 3519 (br, s), 3401 (br, s), 2933 (w), 2667 (w), 2039 (w), 1634 (s), 1432 (m), 1355 (m), 1118 (m), 1041 (m), 1006 (w), 908 (w), 852 (m), 803 (w), 642 (s), 842 (m), 433 (m).

## 3. Results

### 3.1. Structural Description

**Figure 3:**
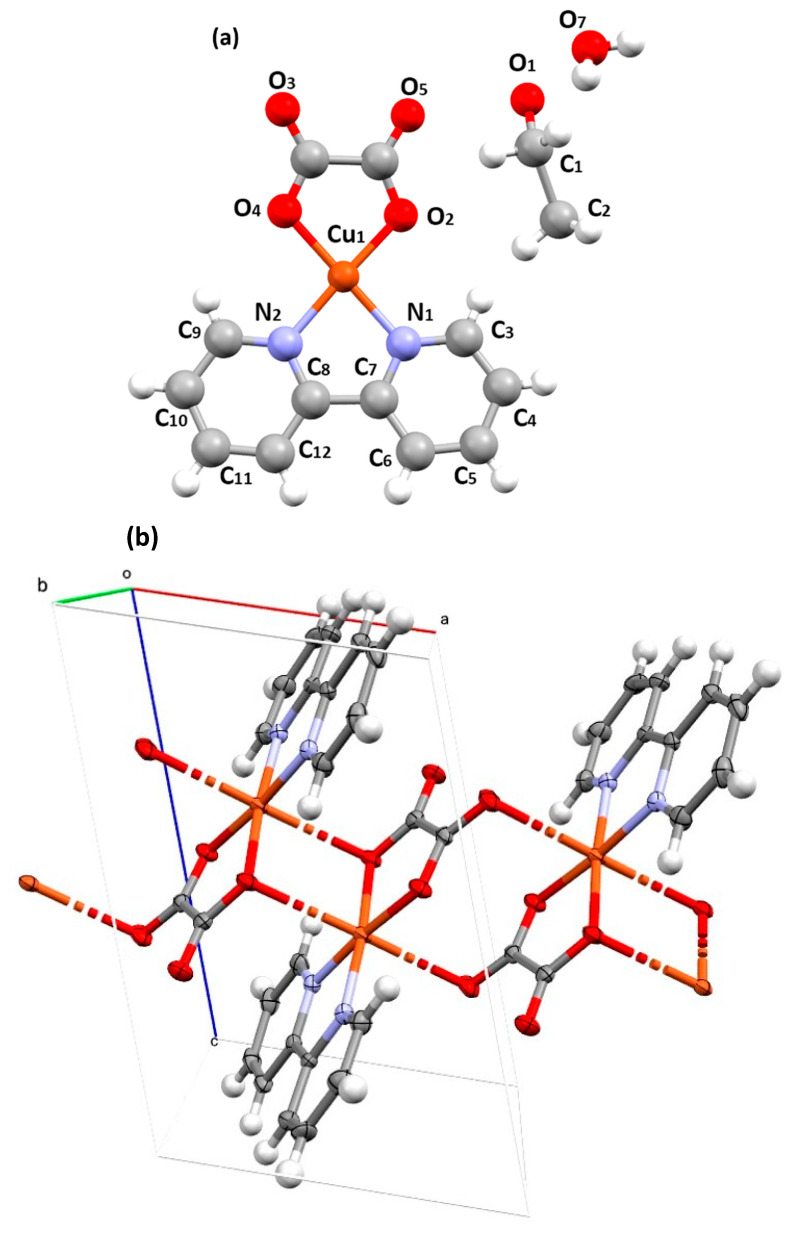
(**a**) Labeled ball-and-stick of the asymmetric unit of **1**; (**b**) ORTEP diagram of **1** showing the polymeric 1D- chain formed along a-axis.

The labeled ball-and-stick of the asymmetric unit for **1** and the ORTEP diagram of the polymeric 1D chain of **1** are represented in Figure 3, as are the asymmetric units of one atom of Cu, 2, 2′, bipyridine, oxalate, and methanol, respectively. Each asymmetric unit is connected with Cu(II) of another unit through the oxygen of oxalate ions to generate a 1D Zig Zag polymer chain. In this regard, oxalate acts as a bridged ligand. Further, one protonated oxalic acid and one water molecule act as counterions. Cu(II) atoms bear six coordination numbers, and their geometry can be best described as octahedral. The coordination around the copper is octahedral, with two oxygens (O2 and O4) from an oxalate and (N1 and N2) from the bipyridine in the equatorial positions, and two other oxygens in the axial positions from oxalates, O2[1 − x,1−y,1 − z] and O3[2 − x,1 − y,1 − z]. Selected distances (Å) and angles (°): Cu_1_-O_2_ 1.931(3), Cu_1_-O_4_ 1.936(3), Cu_1_-N_1_ 1.985(4), Cu_1_-N_2_ 1.964(4), O_2_-Cu_1_-O_4_ 85.2(1), O_2_-Cu_1_-N_1_ 95.9(1), O_2_-Cu_1_-N_2_ 174.7(1), O_4_-Cu_1_-N_1_ 175.0(1), O_4_-Cu_1_-N_2_ 97.2(2), N_2_-Cu_1_-N_1_ 81.2(2), Cu_1_…Cu_1_ (−x, −y, −z) 3.395(1) Å.

A literature search indicated the existence of a few relevant 1D Cu-based oxalate-bridged chains, such as {[Cu(bpy)(ox)]_n_2.5H_2_O}_n_ [46], {[Mn(bpy)(C_2_O_4_)]1.5H_2_O}_n_ [47], and [Cu_2_(μ-C_2_O_4_) (H_2_O)_2_(bpy)_2_(NO_3_)_2_] [48], respectively. Moreover, some other coordination compounds [{Cu(bpy)Cl}_2_(μ-C_2_O_4_)] [49], {Cu(bipy)(bzt)(OH_2_)}_2_(µ-ox)] [50] have been seen with same ligands explored by us in this manuscript. In addition, some other 1D oxalato-bridged compounds with different co-ligands are existed, such as [Cu_2_(µ-ox)_2_(pyz)(µ-pyz)_2_]_n_ [51], [Cu(DPA)(C_4_O_4_)(H_2_O)]_2_·(H_2_O) [52], [Cu(ox)(Im)_2_] [53], {[(CH_3_)_4_N]_2_[Cu(C_2_O_4_)_2_]·H_2_O}_n_ (where [54], {[Cu_4_(ox) (dpp)_2_(Cl)_6_]}_n_ [Cu_4_ (ox) (dpp)_2_ (NO_3_)_6_(H_2_O)_2_]∙1.2(H_2_O)}_n_ [55].

### 3.2. Adsorption Parameter Study

In the current work, compound **1** is explored to adsorb the toxic MB and MO from an aqueous system.

The contact time experiment was performed in order to examine the adsorption ability and removal performance of the Cu compound. For this experiment, the settings are set to pH = 8, MB = 0.01 mg/mL, MO = 0.01 mg/mL, and temperature = 298 K. The adsorption values were taken every 10 min and analyzed. The equilibrium was established approximately in 1 h. The synthesized compound was found to have an adsorption capacity of 194.0 and 167.3 mg/g, and its removal rate was estimated to be 77.6% and 66.9% for MB and MO, respectively. Further, we studied adsorption dynamics using different models, like pseudo-first-order and pseudo-second-order, and the liquid film diffusion model.

### 3.3. Effect of Contact Time

**Figure 4:**
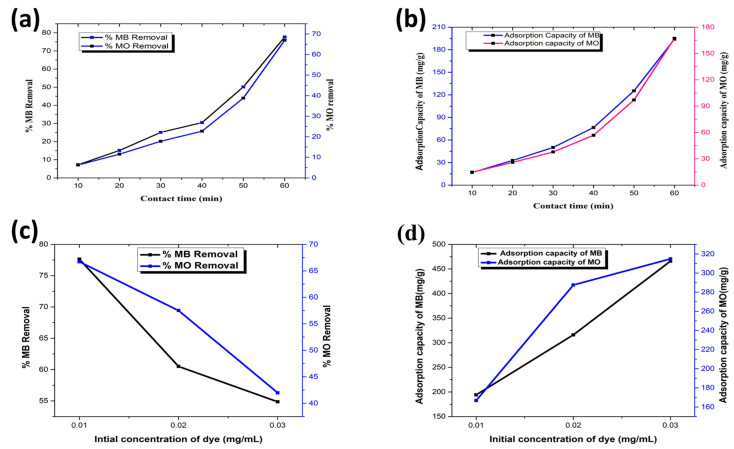
(**a**) Contact time effect on the % age removal efficiency and (**b**) on adsorption ability of dyes; (**c**) Initial concentration effect of dyes on % age removal efficiency and (**d**) on adsorption ability of the dyes.

Contact time is also a crucial variable influencing the rate and amount of material adsorbed during the adsorption process. Using a 0.01 mg/mL dye solution at 298 K and a pH of 8, the impact of contact time on the removal rate and adsorption capacity of MB and MO was examined from 10 to 60 min, as shown in Figure 4a,b. The removal performance increased with the increase in contact time, and a dynamic equilibrium was achieved after a certain time at which the concentration of dye molecules became constant in the solution. The maximum adsorption of MB and MO occurred at t = 60 min. In the beginning, a huge number of active sites were available; therefore, the increase in adsorption capacity was analyzed. A gradual decrease in the rate of adsorption was observed owing to the filling of active sites. Upon reaching the saturation point, no vacant sites were left for the abstraction of MB and MO molecules on the surface as in the published compound [56].

### 3.4. Effect of Initial Concentration

The initial concentration of dye is another crucial factor that determines the extent of the adsorption process. The effect of starting dye variable concentrations from 0.01 to 0.03 mg/mL under the optimum conditions of other experimental parameters (adsorbent concentration 10 mg, pH of 8, with an exposure time of 60 min) was examined in order to evaluate the effectiveness of MB and MO molecules uptake on the adsorbent surface. Figure 4c,d shows that with the increase in starting concentrations of MB and MO, the % removal decreased from 77.60 to 54.8 and 66.7 to 41.9, respectively. This phenomenon may be explained in the following way: The number of adsorption active centers was enough to accommodate MB and MO molecules at low concentrations, leading to an increase in % removal. On the other hand, at higher initial dye concentrations and the same amount of adsorbent, there is a shortage of adsorption capacity [57]. MB, being a cationic dye, shows more adsorption capacity than MO, which is because positive charge density is localized on the entire molecule. The electrostatic interaction (cationic dye) and specific polar group of **1** also play important roles in the adsorption phenomenon. Furthermore, the π-π interaction also paves the way for the stickiness of dyes with **1** [58]. MB is cationic (dye), which has an electrophilic character and interacts with the nucleophilic aromatic ring, as well as anionic (oxalate) from **1,** which is electrostatic interaction results in an enhanced adsorption process. The OH-π interaction is due to the OH of water from the coordination polymer and the π electrons of the MB and MO rings. π-π interactions arise due to the aromatic ring of the bpy and the ring of the dye molecule.

### 3.5. Effect of pH

**Figure 5:**
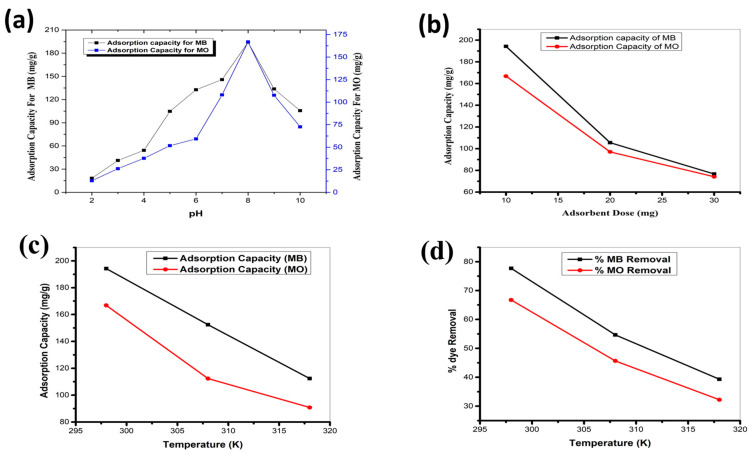
(**a**) Effect of pH and (**b**) effect of adsorbent on adsorption capacity of dyes; (**c**) Effect of temperature on adsorption capacity; and (**d**) on % age efficiency of dyes.

From the results, it is clear that with increasing pH, the capacity of adsorption increases, and the maximum adsorption is noted at a pH of 8.0, with the best removal rates of 77% and 66%, respectively, as shown in Figure 5a. The removal rate of **1** for MB and MO were tested at pH values of 2, 3, 4, 5, 6, 7, and 8. The maximum efficiency is shown at a pH of 8.0, and the best removal rates are 77.6% and 66.9%, respectively. The fabricated material has extensive capability due to its large surface area, pore size, and large number of sites for adsorption. The low adsorption effectiveness exhibited in a more acidic environment is because of structural changes in the adsorbent molecule. The synthesized compound shows better results at different pHs for MB than for MO. The electrostatic interaction between MB and the adsorbent is produced due to a negative charge on the adsorbent surface, resulting in excellent adsorption at pH 7 and 8. Furthermore, due to the existence of H^+^ ions competing with cationic species (MB) in acidic conditions, reduced adsorption efficiency was found. In the case of MO (anionic dye), the adsorbent surface was positively charged in an acidic environment, and the adsorption of this dye was caused by its electrostatic force. MO exhibited superior adsorption in acidic conditions [59].

### 3.6. Effect of Adsorbent Dose

The (**1**) adsorbent dose was increased from 10 to 30 mg for MB and MO adsorption (10 ppm or 0.01 mg/mL), and the adsorption ability and removal performance were evaluated through Equations (1) and (2). Adsorption capacity was reduced from 194.24 to 76.75 mg/g and 167.3 to 74.1 mg/g, respectively, but removal efficiency was raised by increasing the adsorbent dose from 77.7 to 92% and 66.7 to 88.9%, respectively. Figure 5b shows the findings of adsorption capacity and removal efficiency for dyes. Because adsorbent dose is inversely related to adsorption capacity, the adsorption capacity of **1** is decreased with increasing adsorbent dose [60,61].

### 3.7. Effect of Temperature

Temperature has a significant impact on the process of adsorption. Figure 5c,d depicts the temperature effect on dye adsorption. The results show that increasing the temperature reduced the removal performance and adsorption ability because dye adsorption on (**1**) is an exothermic process. 

### 3.8. Adsorption Kinetics

**Figure 6:**
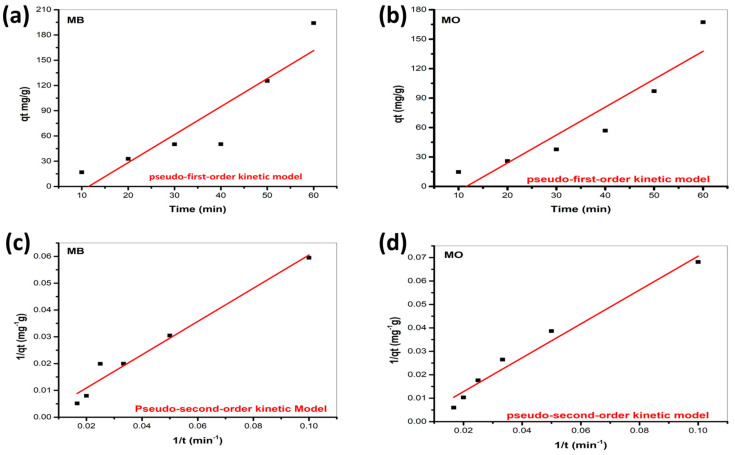
(**a**) Pseudo-first-order kinetic model fitted for MB; (**b**) pseudo-first-order kinetic model fitted for MO; (**c**) pseudo-second-order kinetic model fitted for MB; (**c**) pseudo-second-order kinetic model fitted for MB.

The various kinetic models, like the pseudo-first-order kinetic model and the pseudo-second-order kinetic model, are used to study how MB and MO stick to the complex. The graphical representation of pseudo-first-order kinetic model for MB and MO shown in the Figure 6a,b while pseudo-second-order kinetic model for MB and MO shown in Figure 6c,d respectively. The correlation values of the models are also figured out. The value of R^2^ is the maximum for the pseudo-first-order kinetic model (R^2^ = 0.891), so this model was explored for MB and MO adsorption on (**1**) adsorbent. This shows that physisorption is part of finding the rate at which the complete process is going. This model shows that physical adsorption which is caused by forces that are created when metal ions and polar groups of dye interact [62]. 

### 3.9. Adsorption Isotherm

**Figure 7:**
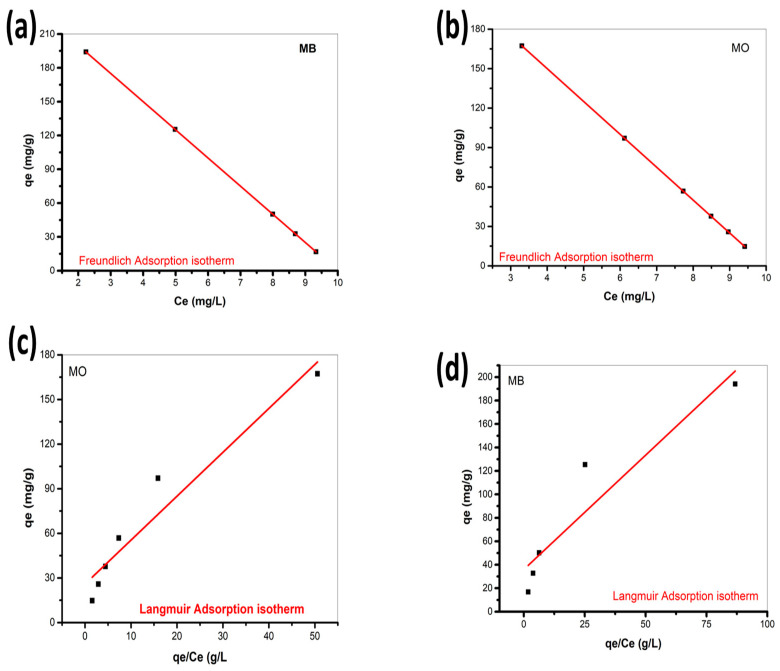
(**a**) Freundlich adsorption isotherm fitted for MB; (**b**) Freundlich adsorption isotherm fitted for MO; (**c**) Langmuir adsorption isotherm fitted for MB; (**d**) Langmuir adsorption isotherm fitted for MO.

For the study of MB and MO adsorption, the various adsorption isotherm models, like the Freundlich isotherm and the Languir isotherm, were examined. Adsorption equilibrium data are used to work with these models. The straight plot of the Freundlich adsorption model for MB and MO is shown in Figure 7a,b while the Langmuir adsorption model is shown in Figure 7c,d respectively. The data make it clear that the Freundlich model has a high correlation coefficient in comparison to the Langmuir model, which shows multi-layer adsorption occurred. So, the best way to explain how MB and MO stick to this copper compound is with the Freundlich adsorption isotherm model, and hence, mono-layered adsorption occurs [63].

### 3.10. Adsorption Mechanism

**Scheme 1:**
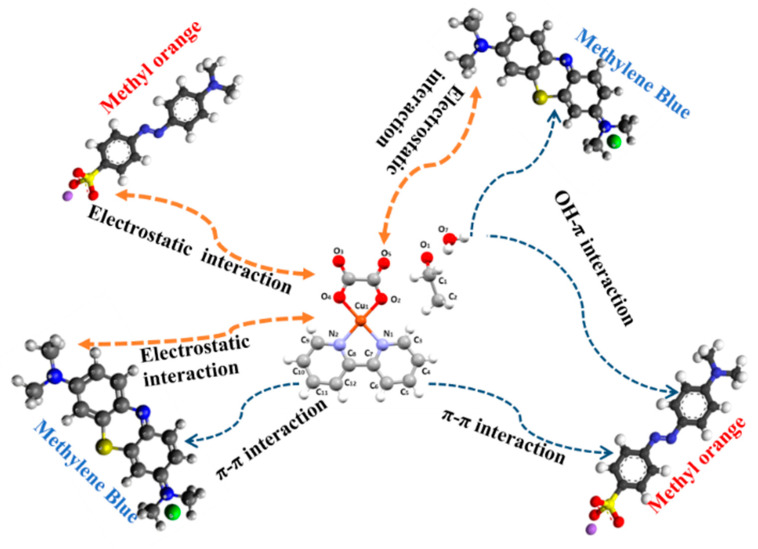
Possible mechanism of cationic/anionic dye adsorption for **1**.

Adsorption is the surface process in which the structure and behavior of the adsorbate compound are examined to determine the adsorption capacity of the adsorbate molecule [64]. In the whole adsorption process, different mechanism possibilities like hydrogen bonds, π-π interactions, and electrostatic interactions may exist [65,66]. The possible mechanisms for the adsorption of **1** and the dyes MB and MO are represented (Scheme 1).

It seems the various possible interactions, such as electrostatic, H-bonding, and π-π, contribute efficiently. The MB^+^ (cationic dye), which is electron-deficient, interacts with nucleophilic aromatic rings of bipyridine through the generation of an electrostatic force of attractions and facilities in adsorption. In addition, MB (cationic dye) and bpy create a π-π interaction that also supports adsorption. In the case of MO^−^ (anionic dye), the electrostatic repulsive force produced between the negatively charged sulfate and nucleophilic aromatic bpy decreases the adsorption process, but MO and hydrogen of the bpy create weak hydrogen bonding and π-π interactions between the aromatic rings of dyes and **1** that facilitate the adsorption of MO. The correlation coefficient has the highest values (R^2^ = 0.9681) and (R^2^ = 0.9617) for MO and MB, respectively. It is noticed in the reported literature that transition metal ions containing 1D coordination polymers have high adsorption for MB in comparison to MO, and their comparison is depicted in Table S3 (Supplementary Materials). 

## 4. Reusability

**Figure 8:**
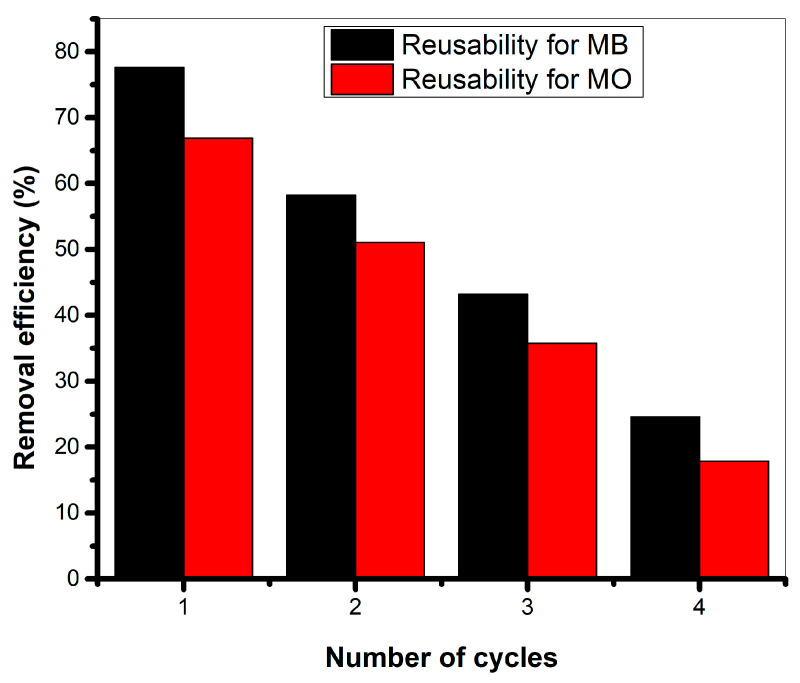
Recyclability of adsorbent **1.**

From an application perspective, one of the most crucial parameters for treating wastewater is the recyclability of the synthesized material. After dye adsorption, **1** was repeatedly rinsed with ethanol solvent until all of the dye molecules were gone, at which point it was reused for the following adsorption step **1** and shows exceptional removal efficiency for MB as compared to MO. This is because of the favorable environment for the adsorption of MB. It has been noted in those four cycles, as shown in Figure 8, that the efficiency decreases after every cycle. This results from a decrease in the quantity of open sites that are accessible for adsorption.

### Liquid Film Diffusion Model

**Figure 9:**
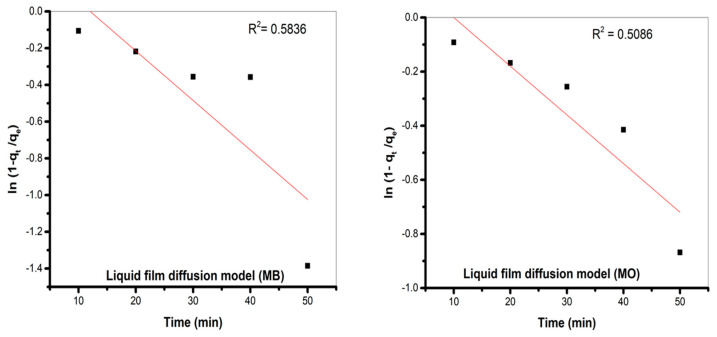
Liquid film diffusion kinetic model fitting.

Assuming that the slowest stage in the process of adsorption is the one that demonstrates the kinetics of the rate processes, the liquid film diffusion model describes the flow of the adsorbate molecules via liquid film surrounding the adsorbent. The following equation represents the liquid film diffusion model [67].

$$\ln\left(1-F\right)=-K_{fd}.t$$

where F = fractional attainment of equilibrium (F = qt/qe), and K_fd_ (min^−1^) = film-diffusion rate coefficient. Figure 9 demonstrated that the experimental MB and MO adsorption data by **1** from an aqueous solution at varying temperatures could not be well-fitted through the liquid film diffusion model, which also produced very low correlation coefficients and poor convergence.

## 5. Conclusions

A 1D Zig-Zag Cu(II) polymer chain is derived from oxalic acid and 2, 2-bipyridine under solvothermal conditions. The prepared compound was explored for the adsorption of toxic cationic (MB) and anionic (MO) dyes in an aqueous system and estimated their adsorption capacities as 77.6% and 66.9% for these dyes, respectively. The adsorption process occurs through pseudo-first-order, and possible mechanisms for adsorption are electrostatic, π-π, and H-bonding interactions. The present study will be useful for coordination researchers to promote the adsorption proficiency of low-cost polymer materials in wastewater treatment for future research.

## Data Availability

Data are contained within the article.

## Supplementary Materials

The following supporting information can be downloaded at: https://www.mdpi.com/article/10.3390/polym16121742/s1, Table S1. Some important crystallographic parameters in **1**. Table S2: Bond distances (Å) and angles (degree) in **1**. Figure S1: TGA Curve for **1**. Table S3: Comparison of reported 1D coordination polymers with our compounds for adsorption of MB and MO.
