# Supplementary material for: An Oxalato-Bridged Cu(II)-Based 1D Polymer Chain: Synthesis, Structure, and Adsorption of Organic Dyes
Source: Polymers (Basel). 2024 Jun 19;16(12):1742. doi: 10.3390/polym16121742 (PMC11207973; doi:10.3390/polym16121742)
Supplement: Supplementary file 1 [file polymers-16-01742-s001.zip › polymers-2977559-supplementary.pdf]

Supplementary Materials

An Oxalato-Bridged Cu(II)-Based 1D Polymer Chain: Synthesis, Structure, and Adsorption of Organic Dyes

Fouzia Munawar [1], Muhammad Khalid [1,*], Muhammad Imran [2], Muhammad Naveed Qasim [2], Shazia Waseem [2], Murad A. AlDamen [3], Muhammad Ashfaq [4], Muhammad Imran [5,6] and Muhammad Nadeem Akhtar [2,*]

[1] Department of Chemistry, Khwaja Fareed University of Engineering & Information Technology, Rahim Yar Khan 64200, Pakistan; fouziamunawar5@gmail.com
[2] Division of Inorganic Chemistry, Institute of Chemistry, The Islamia University of Bahawalpur, Bahawalpur 63100, Pakistan; muhammad.imran@iub.edu.pk (M.I.); n.qasim5122@gmail.com (M.N.Q.); w.shazia@yahoo.com (S.W.)
[3] Department of Chemistry, School of Science, The University of Jordan, Amman 11942, Jordan; maldamen@ju.edu.jo
[4] Department of Physics, University of Sargodha, Sargodha 40100, Pakistan; ashfaq.muhammad@uos.edu.pk
[5] Research Center for Advanced Materials Science (RCAMS), King Khalid University, P.O. Box 9004, Abha 61413, Saudi Arabia; imranchemist@gmail.com
[6] Chemistry Department, Faculty of Science, King Khalid University, P.O. Box 9004, Abha 61413, Saudi Arabia
* Correspondence: muhammad.khalid@kfueit.edu.pk (M.K.); nadeemchem@yahoo.com or m.nadeemakhtar@iub.edu.pk (M.N.A.)

Index

Table S1. Some important crystallographic parameters in 1.
Table S2: Bond distances (Å) and angles (degree) in 1.
Figure S1: TGA Curve for 1.
Table S3: Comparison of reported 1D coordination polymers with our compounds for adsorption of MB and MO.
References

Table S1. Some important crystallographic parameters in **1**.

**Empirical formula**	$\mathbf{C_{13.34}H_{13.33}CuN_2O_{5.67}}$
Formula weight	355.79
Temperature/K	293.15
Crystal system	triclinic
Space group	P_{-1}
a/Å	6.9780(15)
b/Å	9.731(2)
c/Å	10.398(2)
α/°	86.314(7)
β/°	71.369(7)
γ/°	87.927(7)
Volume/$Å^3$	667.5(2)
Z	2
$\rho_{calc}g/cm^3$	1.770
μ/mm^{-1}	1.667
F(000)	363.0
Crystal size/mm^3	0.273 × 0.1 × 0.068
Radiation	MoKα (λ = 0.71073)
2Θ range for data collection/°	5.728 to 50.052
Index ranges	-8 ≤ h ≤ 8, -11 ≤ k ≤ 11, -12 ≤ l ≤ 12
Reflections collected	21234
Independent reflections	2350 [R_{int} = 0.0830, R_{sigma} = 0.0400]
Data/restraints/parameters	2350/0/214
Goodness-of-fit on F^2	1.152
Final R indexes [I>=2σ (I)]	R_1 = 0.0499, wR_2 = 0.1236

Table S2: Bond distances (Å) and angles (degree) in **1.**

**Bond distance (Å)**		**Length/Å**	
**Cu1**	O2	1.931(3)	
**Cu1**	O4	1.936(3)	
**Cu1**	N1	1.985(4)	
**Cu1**	N2	1.964(4)	

**Bond angles (degree)**			**Angle/°**	
**O2**	Cu1	O4	85.16(13)	
**O2**	Cu1	N1	95.91(14)	
**O2**	Cu1	N2	174.73(14)	
**O4**	Cu1	N1	175.03(14)	
**O4**	Cu1	N2	97.18(15)	
**N2**	Cu1	N1	82.17(15)	

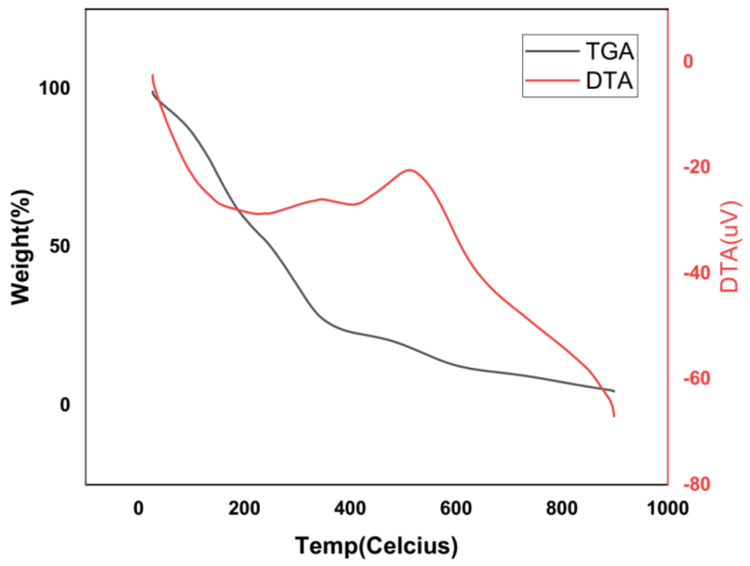

Figure S1: TGA Curve for **1.**

Table S3: Comparison of reported 1D coordination polymers with our compounds for adsorption of MB and MO.

**Materials**	**$Q_o$max, (mg g-1)**	**Adsorption Kinetic model**	**Mechanism**	**Ref**
$[\{Co(pza)_2(SCN)_2\}(pza)]_n$	**MB > MO**	Pseudo second order kinetic model	Electrostatic interaction, π–π interaction	[1]

$[Zn_2(L)_2(bpe)_2(H_2O)_2]$	**MB** > **MO**	Pseudo second order kinetic model	Electrostatic interaction, π–π interaction	[2]
$[Ni(Hpdc)(H_2O)(\mu\text{-}H_2O)]_n$	**MB >** **MO**	Pseudo-second-order kinetic model	Electrostatic interaction, π–π interaction	[3]
$[Ni(pyc)_2(H_2O)]_n$	**MB** > **MO**	Pseudo-second-order kinetic model	Electrostatic interaction, π–π interaction	[3]
**1**	**MB** > **MO**	Pseudo-second-order kinetic model	Electrostatic interaction, π–π interaction	This work

Abbreviations: pza=pyrazine carboxamide;
L = 4,4'-((1,2- phenylenebis(methylene))bis(oxy))dibenzoic acid;
bpe = (E)-1,2-di(pyridin-4-yl)ethane;
Hpdc = 3,5-pyrazoledicarboxylic acid;
pdc = pyrazine-2-carboxylic acid

References

1. Ahmad, M. S.; Khalid, M.; Khan, M. S.; Shahid, M.; Ahmad, M., Exploiting one dimensional polymer for environmental monitoring: Co based coordination polymer for efficient removal of cationic dyes. *Journal of Solid State Chemistry* **2022,** *313*, 123307.

2. Hu, F.-l.; Shi, Y.-X.; Chen, H.-H.; Lang, J.-P., A Zn(II) coordination polymer and its photocycloaddition product: syntheses, structures, selective luminescence sensing of iron(III) ions and selective absorption of dyes. *Dalton Transactions* **2015,** *44* (43), 18795-18803.

3. Ahmad, M. S.; Khalid, M.; Khan, M. S.; Shahid, M.; Ahmad, M., Ni(III)-Based one dimensional coordination polymers for environmental remediation: design, topology, magnetism and the selective adsorption of cationic dyes. *CrystEngComm* **2021,** *23* (36), 6253-6266.
